# Identification of early and extra-early maturing tropical maize inbred lines resistant to *Exserohilum turcicum* in sub-Saharan Africa

**DOI:** 10.1016/j.cropro.2020.105386

**Published:** 2021-01

**Authors:** Baffour Badu-Apraku, Faith Ayobami Bankole, Babatope Samuel Ajayo, Morakinyo Abiodun Bamidele Fakorede, Richard Olutayo Akinwale, Abidemi Olutayo Talabi, Ranajit Bandyopadhyay, Alejandro Ortega-Beltran

**Affiliations:** a International Institute of Tropical Agriculture (IITA), Ibadan, Nigeria; b Obafemi Awolowo University, Ile-Ife, Nigeria

**Keywords:** Disease resistance, Germplasm screening, Index selection, Sustainable food production, ASI, anthesis-silking interval, BI, base index, DA, days to 50% anthesis, DS, days to 50% silking, EASP, ear aspect, EHT, ear height, EEM, extra-early maturing, EM, early maturing, EPP, number of ears per plant, G × T, genotype × trait, G × E, genotype by environment, GYLD, grain yield, HUSK, husk cover, IITA, International Institute of Tropical Agriculture, Low N, low soil nitrogen, PASP, plant aspect, PHT, plant height, PSI, percentage severity index, RL, root lodging, SAS, statistical analysis system, SL, stalk lodging, SSA, sub-Saharan Africa, TURC2WAI, disease score 2 weeks after inoculation, TURC6WAI, disease score 6 weeks after inoculation, TURC, average disease severity score, WCA, West and central Africa

## Abstract

Northern corn leaf blight (NCLB) incited by the fungus *Exserohilum turcicum* is a foliar disease that significantly limits maize production and productivity in West and Central Africa (WCA), particularly in the mid-altitudes but during the last decade it has become a menace in lowland agro-ecologies. The most economical and environmentally friendly disease management strategy is the cultivation of maize varieties resistant or tolerant to NCLB. However, no early maturing (EM) and extra-early maturing (EEM) NCLB resistant varieties are commercially available in WCA. One hundred inbred lines each of EM and EEM derived from tropical maize germplasm were inoculated with a virulent isolate of *E. turcicum* at five locations in Nigeria during the 2017 and 2018 growing seasons. The objective of the study was to identify promising NCLB resistant lines and to investigate inter-relationships among the traits. Analysis of variance revealed highly significant genotype and genotype by environment (G × E) interactions for disease severity, grain yield (GYLD), and other agronomic traits. The average disease severity (TURC) values ranged from 1.9 to 5.8 and 2.9 to 5.7 for the EM and EEM inbred lines, respectively. The levels of reaction of the inbred lines to NCLB ranged from highly resistant to highly susceptible. Stepwise regression analysis showed that ears per plant, ear and plant aspects were significantly influenced by the disease scores. Ears per plant, ear and plant aspects, TURC and GYLD traits were employed to develop a base index (BI) for selecting NCLB resistant inbred lines for hybrid development. TZEI 135 and TZEEI 1 were outstanding in GYLD and also had the highest positive BI values in the EM and EEM inbred lines, respectively. The identification of NCLB resistant lines in this study has set the premise for development of NCLB resistant hybrids for WCA as well as the improvement of tropical maize breeding populations for NCLB resistance.

## Highlights

• Protocol for inoculum production and inoculation of maize with *E.* *turcicum* for resistance screening of pathogen developed.
• Early and extra-early maize inbred lines with resistance to *E. turcicum* and adapted to the tropics have been identified.
• A base index for selecting maize inbred lines for resistance to *E. turcicum* with desirable agronomic traits developed in IITA.

## 1 Introduction

A large proportion of the human population in sub-Saharan Africa (SSA), relies on maize as primary staple food crop. Maize harvested at the dough stage of grain-filling, referred to as green maize, may be roasted or boiled with or without the husk while the immature cooked grains are consumed as a snack or partial meal. Also, a variety of traditional meals are produced with milled dry maize grains (Badu-Apraku and Fakorede, 2017). In addition, there is increased preference for maize grains over products of other crops as raw materials for emerging and growing livestock feed and brewery industries (Badu-Apraku et al., 2013; Badu-Apraku and Fakorede, 2017).

Several biotic and abiotic stresses impede the attainment of maximum maize yield potential. Prominent among the stresses is the attack caused by the fungus *Exserohilum turcicum* (Pass.) Leonard & Suggs, which commonly infects maize cultivated in humid mid- and high-altitude regions of the world, including SSA (Sibiya et al., 2013; Hooda et al., 2016). This fungus, which causes the disease known as northern corn leaf blight (NCLB), commonly thrives in mid-altitude tropical regions with 70–95% humidity and temperatures ranging from 17 to 28 °C (Carson, 2007; Ahangar et al., 2016). In West and Central Africa (WCA), the disease was typically restricted to mid- and high-altitudes of Nigeria and Cameroon but has recently spread to lowland areas which were traditionally free of NCLB (Badu-Apraku and Fakorede, 2017; Akinwale and Oyelakin, 2018). NCLB reduces maize grain yield by up to 30% in Southern Africa (Kloppers and Tweer, 2009; Human et al., 2016; Weems and Bradley, 2018), thereby posing a significant threat to food security in SSA, especially in those countries that are not self-sufficient in maize production (Human et al., 2016). For example, Nigeria annually imports over 400,000 tons of maize to satisfy the needs of humans and livestock industries (USDA, 2018). The development of maize cultivars with resistance to NCLB and adaptation to the diverse agro-ecological zones of WCA would reduce losses associated with the disease and contribute to decrease in volumes of maize imports. Breeding for host plant resistance is the most appropriate, environmentally friendly and economical mitigation option for combating most plant diseases (Ayiga-Aluba et al., 2015; Chen et al., 2016; Wiesner-Hanks and Nelson, 2016). Therefore, breeeding maize hybrids with resistance to NLCB offer the most sustainable, environmentally friendly and economically feasible management strategy.

Estimates of heritability are indispensable parameters in maize improvement programs and are usually lower under stress than non-stress conditions, especially for grain yield (Bolaños and Edmeades, 1993; Badu-Apraku et al., 2004; Badu-Apraku et al. 2012). Fortunately, heritability of some secondary traits in maize, such as number of ears per plant (EPP), are relatively higher than that of grain yield under stress environments. Information on inter-relationships among traits has, therefore, been extensively used by breeders in the development of indices for identification and selection of genotypes for resistance/tolerance to stresses (Badu-Apraku and Fakorede, 2017). The information is easily obtained through stepwise regression analysis and sequential path-coefficient analysis. Stepwise multiple regression analysis plays a key role in identifying secondary traits with significant contributions to grain yield (primary trait) while sequential path analysis categorizes secondary traits based on the relative importance of their direct and indirect contributions to grain yield in consequential order (Mohammadi et al., 2003; Talabi et al., 2017).

The International Institute of Tropical Agriculture (IITA) has devoted significant research efforts to the development and commercialization of early maturing (EM; 90–95 days to physiological maturity), and extra-early maturing (EEM; 80–85 days to physiological maturity) maize hybrids (Badu-Apraku and Fakorede, 2017). Availability of both EM and EEM maize hybrids have contributed immensely to the spread and increased adoption of maize into marginal areas (<500 mm annual rainfall) in the lowland savannas of WCA (Badu-Apraku and Fakorede, 2017). In addition, because of the prevalent global climate change effects, rainy season patterns in the rain forest agroecology of WCA have changed with significantly less rainfall in the early part of the season and abrupt cessation at the end of it. This also has encouraged farmers in lowland areas to adopt EM and EEM maize. However, none of the maize varieties adapted to lowlands of WCA have been screened for resistance to NCLB, which is becoming a threat to increased maize production and productivity. It is unknown whether resistance exists among EM and EEM maize germplasm. Recent severe NCLB outbreaks in parts of WCA lowlands calls for the development of *E. turcicum* resistant EM and EEM maize hybrids to pre-empt the chances of future epidemics and subsequent yield losses. To effectively develop high yielding varieties with superior resistance to NCLB in the two maturity groups, information is needed on the genetic variation for NCLB resistance in the available germplasm of WCA, heritability of the resistance, and inter-relationships among NCLB resistance, grain yield, and other desirable agronomic traits.

Use of stress resistant/tolerant parental inbred lines increases the chances of developing stress resistant/tolerant maize hybrids (Badu-Apraku and Fakorede, 2017). Thus, the first step towards the development of outstanding *E. turcicum* resistant or tolerant maize hybrids is the identification of promising parental lines. This study sought to identify EM and EEM maize inbred lines with resistance to NCLB. The objectives of the present study were to i) develop the protocol for *E. turcicum* inoculum production and inoculation, ii) screen selected EM and EEM inbred lines under artificial *E*. *turcicum* infection, iii) classify the inbred lines into heterotic groups based on varying degrees of resistance/tolerance and susceptibility to NCLB, iv) identify and select EM and EEM maize inbred lines with resistance to NCLB, and v) elucidate the inter-relationships among grain yield, NCLB resistance, and other agronomic traits under artificial *E. turcicum* inoculation. Results of our study will facilitate the development of EM and EEM maize hybrids with resistance to NCLB for commercialization in SSA.

## 2 Materials and methods

### 2.1 Fungal isolation

Naturally infected maize plants showing characteristic NCLB symptoms were identified in research fields of IITA in Ibadan, Ikenne, and Ile-Ife, Nigeria. Infected leaves were detached, placed inside labeled paper bags, and immediately taken to the laboratory. Sections of leaves with lesions were cut with a sterile scalpel blade and surface-sterilized with 50% NaOCl for 1 min. The leaf sections were rinsed in three changes of sterile distilled water and blotted free of excess moisture using sterile paper towel inside a biosafety cabinet. Leaf fragments were then plated onto Acidified Potato Dextrose Agar (APDA; 0.15% lactic acid) and incubated for 3 d at 28 °C. Mycelia growing from diseased sections were transferred to APDA plates and incubated for 5 d at 25 °C. Isolates were then single-spored in APDA and incubated for 5 d at 25 °C. Recovered isolates grew as black fluffy mycelia with grey coloured aerial hyphae which spread radially on the medium. Recovered *E. turcicum* isolates were saved as agar plugs of the cultures in 4 ml vials containing 2 ml sterile water and stored at 4 °C.

### 2.2 Pathogenicity tests using a detached leaf assay

Seven *E. turcicum* isolates recovered from Ibadan (3), Ikenne (3), and Ile-Ife (1) were tested for pathogenicity using a detached leaf assay (DLA). The DLA was developed for testing maize genotypes for resistance to *Bipolaris maydis* (Aregbesola et al., 2019). In the present study, we determined the most appropriate *E. turcicum* inoculum concentration under various conditions using the DLA. Briefly, sections of leaves (5 cm^2^) of two EEM inbred lines, TZEEI 3 and TZEEI 30 were placed on 1% Technical Agar (Oxoid, Unipath Ltd., Hampshire, England) amended with 45 ppm 6-benzylamino purine (BAP). Leaf sections were independently inoculated with spore suspensions of the evaluated isolates using three concentrations: 10^4^, 10^5^, and 10^6^ spores ml^−1^ (Table 1). Plates were incubated for 10 d at 25 °C. Leaves were scored for disease severity using a scale of 1–5 where 1 = no visible symptoms; 2 = 1–10% leaf area covered with brownish chlorosis; 3 = 11–25% leaf area covered with brownish leisions; 4 = 26–50% leaf area covered with brownish lesions; and 5 = > 50% leaf area covered with brownish lesions.

**Table 1:** Effect of different inoculum concentrations of *Exserohilum turcicum* on northern corn leaf blight of maize (*Zea mays* L.) using a detached leaf assay.

CONCENTRATION	TURC4DAI	TURC6DAI	TURC8DAI	TURC10DAI
Control	0.1	0.1	0.2	0.2
10^4^	1.0	1.9	3.1	3.8
10^5^	1.2	2.1	3.4	4.2
10^6^	0.8	1.7	2.8	4.0
LSD	0.1	0.1	0.5	0.2
P for Genotype	a	a	a	a
P for G*C	a	a	a	a
CV (%)	30	27	23	18
R^2^ (%)	53	62	71	82

a Significant at *P* < 0.01; TURC: disease severity based on a rating scale of 1–5 where 1 = no visible symptoms; 2 = 1–10% leaf area covered with brownish chlorosis; 3 = 11–25% leaf area covered with brownish leisions; 4 = 26–50% leaf area covered with brownish lesions; and 5 = > 50% leaf area covered with brownish lesions, DAI: days after inoculation, CV: coefficient of variation, R^2^: Coefficient of determination.

### 2.3 Pathogenicity tests using fungal suspensions

The spores of 10-day-old cultures of *E. turcicum* isolate NGIB16-13 grown on APDA were washed with 1% sterile TWEEN 20® and aseptically diluted to 10^5^ spores ml^−1^. Leaves of 21-day-old maize plants were sprayed with the fungal suspension until run-off. Control plants were inoculated with sterile distilled water. The inoculated plants were covered with clean nylon bags for 48 h to create a humid environment favourable for pathogen establishment and disease development. Disease severity was determined using the 1 to 5 scale described earlier.

### 2.4 Pathogenicity tests using colonized sorghum grains as inoculum

Fifty grams of white sorghum were placed in 250 ml clean Erlenmeyer flasks and soaked overnight with 200 ml tap water. After 24 h, water was decanted, flasks were carefully covered with cotton wool and aluminum foil, and autoclaved at 121 °C for 1 h. Spores from 14-day-old cultures of *E. turcicum* isolate NGIB16-13 were harvested and adjusted to a concentration of 10^5^ spores ml^−1^, as described earlier. Flasks containing the sterilized sorghum were aseptically inoculated with 4 ml of spore suspension. Inoculated flasks were shaken thoroughly for even distribution of the spore suspension. Flasks were incubated at room temperature for 5 d, on an unilluminated laboratory bench. The flasks were shaken at 24, 72, and 96 h after inoculation to allow for even colonization. After the incubation period, the colonized grains were stored in a refrigerator (4 °C) and later used for the inoculations.

The whorl of 21-day-old maize plants was inoculated with three colonized sorghum grains and covered with clean nylon bags for 48 h. Control plants were inoculated with sterile, non-inoculated sorghum. Four plants from two pots were evaluated for disease severity using the 1 to 5 scale described earlier.

### 2.5 Inoculum preparation for field studies

One hundred grams of white sorghum were pre-conditioned and autoclaved as described above. Inoculum production for each location was done using a total of 100 Erlenmeyer flasks. A spore suspension of *E. turcicum* isolate NGIB16-13 was obtained and diluted as described earlier. Each flask was inoculated with 4 ml of spore suspension. Incubation and shaking of the inoculated flasks were done as described earlier. Colonized sorghum grains without any sign of contamination were stored in a refrigerator (4 °C) until transported to fields for inoculation. Inoculum was stored in a refrigerator for a maximum of 2 d.

### 2.6 Field inoculation and screening of inbred lines

One hundred each of EM (50 white, 50 yellow endosperm) and EEM (50 white, 50 yellow endosperm) maize inbred lines (Supplementary Tables 1 and 2) were planted at Ikenne (6°53′ N, 3°42′ E) and Ile-Ife (7°18̕’ N, 4°33̕’ E) in 2017, and Ikenne, Ile-Ife, and Zaria (11°7̕’ N, 7°45̕’ E) during the 2018 growing seasons. The characteristics of the test locations are described in Table 2. Maize planted in those locations which match the target testing environments of the Maize Improvement Programme (MIP) of IITA and surrounding areas have recently experienced severe NCLB outbreaks.

**Table 2:** Characteristics of locations where early and extra-early maturing maize inbred lines were artificially inoculated with *Exserohilum turcicum* during 2017 and 2018.

Location	Coordinates	Agro-ecological zone	Elevation (m)	Average humidity (%)	Annual rainfall (mm)	Average temperature (°C)
Ikenne	6°53′ N, 3°42′ E	Rain Forest	60	81	1800	26
Ile-Ife	7°18̕’ N, 4°33̕’ E	Rain Forest	280	81	1600	25
Zaria	11°7̕’ N, 7°45̕’ E	Northern Guinea Savanna	640	68	1500	27

Randomizations were restricted to each of the maturity groups, which were treated as independent experiments, each laid out as 10 × 10 lattice with 2 replications at all test locations. In the experiments, each inbred line (entry) was planted in a 4 m single-row plot with a spacing of 0.75 × 0.40 m. Three seeds were planted per hill and emerged plants were thinned to two per hill at 2 weeks after planting, resulting in 22 plants per plot. NPK 15-15-15 fertilizer was applied at 3 weeks after planting while urea was top-dressed at 5 weeks after planting. Maize plants within each plot were artificially inoculated at 4 weeks after planting by placing 10 to 15 *E. turcicum* colonized sorghum grains (at a rate of 40 kg/ha) into the maize whorl using a sterile scoop calibrated to deliver the same amount of inoculum per plant. The inoculum concentration for field evaluations was higher than in the preliminary pathogenicity test in order to expose the inbred lines to high pathogen pressure. A total of 8800 plants were manually inoculated at each location (4400 plants per maturity group) during each cropping season for the two years.

### 2.7 Disease scoring

Whole plots of each of the two experiments at the five locations were visually scored twice for disease severity: i) two weeks after inoculation (TURC2WAI, 42 days after planting) to determine the initial response of the inbred line at the early growth stage and, ii) six weeks after inoculation (TURC6WAI, 70 days after planting) to determine the reaction of the inbred lines as they mature. It is known that resistance to a pathogen in plants could improve as the plant matures (Vivek et al., 2013). It would then be valuable to determine whether an initially susceptible inbred line (at the first scoring) could have improved in resistance as it matured or a resistant genotype could have succumbed. Scores were done using the scale of 1–9 adapted from Ramathani et al. (2011) (Supplementary Table 3). For each inbred, the average of the two evaluations was calculated and was designated as TURC. The TURC values were used to classify the inbred lines as highly resistant (1.0–2.4), resistant (2.5–3.4), moderately resistant (3.5–4.4), susceptible (4.5–5.4), and highly susceptible (5.5–9.0).

### 2.8 Measurement of other agronomic traits

The day in which 50% of plants in a plot were shedding pollen was recorded as number of days from planting to anthesis (DA). Number of days from planting to incipient silk (DS) extrusion was similarly obtained. Anthesis-silking interval (ASI) was computed as the difference between DS and DA. Plant height (PLHT) and ear height (EHT) were measured as the distance from the base of the plant to the height of the first-tassel branch and to the node bearing the upper ear, respectively. Root lodging (RL) was calculated as number of plants leaning more than 30° from the upright position. Number of stalks broken below the ear before harvesting was recorded as stalk lodging (SL). Number of ears per plant (EPP) was obtained by dividing the total number of ears per plot by the number of plants harvested. Plant aspect (PASP) was scored based on the overall plant appeal, considering factors such as relative uniformity of PHT and ear placement, uniformity, reaction to diseases and insects, and lodging, using a scale of 1–9 where 1 = excellent phenotypic appeal and 9 = poor phenotypic appeal. Ear aspect (EASP) factored extent of disease and insect damage observed in ears, ear size, and uniformity. The EASP was rated on a scale of 1–9 where 1 = clean, uniform, large, and well-filled ears and 9 = ears with undesirable features. Husk cover (HUSK) was scored on a scale of 1–9 where 1 = husks tightly arranged and extended beyond the ear tip and 9 = open tip cover (ear tips exposed). The rating scales used for PASP, EASP, and HUSK have been described in detail (Badu-Apraku et al., 2012a, Badu-Apraku et al., 2012b) and presented in Supplementary Tables 4, 5 and 6. The traits are routinely scored by breeders and experienced field staff of the IITA-MIP.

Field weight was the weight in kg of all de-husked ears in the plot. The GYLD in kg ha^−1^ was calculated based on 80% shelling percentage, adjusted moisture content of 15%, which was computed by the formula reported by Badu-Apraku et al., 2012a, Badu-Apraku et al., 2012b:

Grain yield (kg ha^−1^) = Field weight (kg) × (100 – actual grain moisture %)/85 × {10,000/plot area (m^2^)} × 0.80.

### 2.9 Data analysis

Data on GYLD, disease score (subjected to square root transformation), and other agronomic traits weresubjected to analysis of variance (ANOVA) using SAS version 9.13 (SAS Institute, 2011). Fisher's protected least significant difference (LSD) test (α = 0.05) was used to separate the means. The mean phenotypic values of all traits were subjected to correlation, stepwise regression, and sequential path analyses using SAS to investigate associations among traits. A genotype × yield × trait (GYT) biplot was used to further elucidate inter-relationships between and among traits and identify inbred lines that were outstanding for each of the measured traits using the ‘which-won-where’ view of the biplot. The genotpes on the vertices of the biplot polygon have the largest value for G × Y × T combinations within each sector of the biplot (Yan and Frégeau-Reid, 2018).

A base index (BI) was developed by determining which trait(s) contributed significantly to grain yield reduction due to infection by *E. turcicum* using stepwise regression and correlation analyses. The BI facilitated the selection of outstanding inbred lines that combined high grain yield and resistance to the pathogen for development of resistant high yielding hybrids for further testing and commercialization in SSA. The correlation coefficients (r-values) of the identified traits were used as the economic weights and were assigned to each trait in the BI, with 1 assigned as the economic weight for grain yield. The BI was developed for each maturity group using the following model:

$$BI=GYLD+\sum_{i=1}^{n}riXi$$

where BI = base index value; X_i_ = trait identified from the regression analysis to have a significant relationship and contribution to GYLD; *i* = 1,2, … *n* traits; and r = correlation coefficient between GYLD and X_i_. BI values were computed for each inbred line. Each parameter of the BI was standardized (with mean = 0 and standard deviation = 1) to minimize the effects of different scales. A positive BI value therefore indicated that a line possesed resistance to *E. turcicum* infection while inbred lines with negative BI values were susceptible to the pathogen.

As proposed by Mohammadi et al. (2003), the predictor traits were organized as first, second, and third-order traits based on their relative importance and contributions to the variation in the dependent variable, GYLD. The procedure of the sequential path analysis used to determine the inter-trait relationships has been described in detail (Badu-Apraku et al., 2012a, Badu-Apraku et al., 2012b; Talabi et al., 2017). Path co-efficients are standardized partial regression co-efficients obtained from stepwise multiple regression analysis and were used for the path analysis to determine inter-trait relationships. Briefly, GYLD was initially regressed on all other measured traits and the traits with significant direct contributions to GYLD were regarded as first order traits. Thereafter, the second order traits were identified by excluding the first order traits and regressing each of the first order traits on the remaining traits. This procedure was repeated until all significant relationships were exhausted at α = 0.05.

## 3 Results

### 3.1 Fungal isolation and preliminary pathogenicity tests

The fungi isolated from all examined infected maize leaf sections had identical morphological features of conidia, conidiophores, and hyphae for *E. turcicum* (Tang et al., 2015; Carson, 2016). The mycelia of the colonies were black and grew in a circular fashion and also had grey aerial hyphae. Conidia of the recovered fungi were pale brown, long, and spindle-shaped with 2–13 cross septa. There was a protruded hilum at one end of all conidia, which is characteristic of *E. turcicum* (Supplementary Figure 1). There were seven different isolates obtained from Ibadan, Ikenne, and Ile-Ife locations. The isolates were named as NGIB16-2, NGIB16-3, NGIB16-13, NGIK16-5, NGIK16-11, NGIK16-12, and NGIF16-6.

Re-inoculation of the seven isolates on healthy maize tissues caused NCLB symptoms (Supplementary Figures 2 and 3). From the seven examined isolates, NGIB16-13 was detected to be the most virulent in DLA and screenhouse tests (under both atomized suspensions and inoculated sorghum grains (Supplementary Figures 2 and 4). In the DLA, 10^5^ spores ml^−1^ was identified as the most appropriate spore concentration for assessment of an *E. turcicum* isolate's pathogenicity (Table 1).

### 3.2 Quality of the inoculum produced for field experiments

Sorghum grains of all flasks were completely colonized by *E. turcicum* isolate NGIB16-13 and there were no signs of contamination by other organisms. There were no visual differences in the quality of inoculum produced for the different locations, during the two years.

### 3.3 Field evaluation of the maize inbred lines

The results of the combined analysis of variance (ANOVA) is presented in Supplementary Tables 7 and 8 and revealed significant mean squares for inbred lines (G), disease severity scores, grain yield, and other measured agronomic traits. Also, there was a highly significant G × E interaction mean squares for disease severity scores, grain yield, and other agronomic traits. Disease severity scores during the first evaluation ranged from 1.9 to 5.9 for EM and from 3.0 to 6.1 for EEM inbred lines (Table 3, Table 4). Similar trends were detected during the second evaluation with 2.4–6.9 for EM and 3.5 to 6.6 for EEM inbred lines.

**Table 3:** Disease severity score of selected early maturing inbred lines artificially inoculated with *Exserohilum turcicum* at Ikenne and Ile-Ife in 2017 and Ikenne, Ile-Ife, and Zaria in 2018.

Entry no.	Genotype	TURC2WAI^a^		TURC6WAI^a^	TURC^b^		Reaction^c^
24	TZEI 60	1.9		2.4	2.0		HR
44	TZEI 144	1.8		2.9	2.2		HR
67	TZEI 122	2.6		2.4	2.3		HR
19	TZEI 53	2.2		3.1	2.4		HR
76	TZEI 135	2.6		2.8	2.4		HR
28	TZEI 75	2.2		3.2	2.5		R
8	TZEI 33	3.0		2.7	2.6		R
78	TZEI 138	2.8		2.9	2.6		R
83	TZEI 146	3.0		2.8	2.6		R
56	TZEI 14	2.3		3.4	2.6		R
41	TZEI 94	3.2		3.5	3.0		R
12	TZEI 45	3.3		3.5	3.1		R
57	TZEI 15	2.8		4.0	3.1		R
4	TZEI 5	3.2		3.8	3.2		R
34	TZEI 86	3.8		4.0	3.5		MR
38	TZEI 90	4.2		4.9	4.2		MR
70	TZEI 127	4.2		5.1	4.2		MR
23	TZEI 59	5.1		6.2	5.1		S
32	TZEI 82	5.1		6.9	5.5		HS
81	TZEI 142	5.9		6.8	5.8		HS
LSD		0.7	0.8			0.6	
RANGE		1.9 to 5.9	2.4 to 6.9			1.9 to 5.8	

a WAI: weeks after inoculation.

b WAF: weeks after flowering.

c HR: highly resistant; R: resistant; MR: moderately resistant; S: susceptible; HS: highly susceptible.

**Table 4:** Disease severity score of selected extra-early maturing inbred lines artificially inoculated with *Exserohilum turcicum* at Ikenne and Ile-Ife in 2017 and Ikenne, Ile-Ife, and Zaria in 2018.

Entry no.	Genotype	TURC2WAI^a^	TURC6WAI^a^	TURC^b^	Reaction^c^
129	TZEEI 32	3.0	3.5	2.9	R
114	TZEEI 15	3.4	3.3	3.0	R
144	TZEEI 48	3.2	3.5	3.1	R
125	TZEEI 28	3.2	3.6	3.1	R
141	TZEEI 45	3.7	3.3	3.1	R
108	TZEEI 8	3.2	3.8	3.1	R
188	TZEEI 99	3.4	4.0	3.4	R
124	TZEEI 27	3.3	4.0	3.4	R
172	TZEEI 79	3.5	3.9	3.4	R
199	TZEEI 172	3.7	3.8	3.4	R
147	TZEEI 51	3.7	4.2	3.6	MR
119	TZEEI 21	3.8	4.3	3.7	MR
161	TZEEI 67	4.0	4.3	3.7	MR
103	TZEEI 3	3.4	4.7	3.7	MR
119	TZEEI 1	3.9	4.4	3.7	MR
157	TZEEI 63	4.8	5.8	4.8	S
136	TZEEI 40	4.8	5.9	4.9	S
173	TZEEI 80	5.5	6.6	5.5	HS
192	TZEEI 108	5.7	6.6	5.6	HS
122	TZEEI 25	6.1	6.6	5.7	HS
LSD		0.8	0.7	0.54	
RANGE		3.0 to 6.1	3.5 to 6.6	2.9 to 5.7	

a WAI: weeks after inoculation.

b TURC: Average disease severity score.

c HR: highly resistant; R: resistant; MR: moderately resistant; S: susceptible; HS: highly susceptible.

Mean values for BI, grain yield, and other measured traits of the selected EM and EEM inbred lines (top 15 and worst 5) are presented in Table 5, Table 6. For EM inbred lines, TZEI 135 and TZEI 60 were the highest yielding while TZEI 142 was the lowest (Table 5). The EEM inbred lines, TZEEI 1, TZEEI 21, and TZEEI 32 were the highest yielding and were significantly different from each other (*P* < 0.05) (Table 6). Overall, 53% and 55% of the EM and EEM inbred lines had positive BI values, respectively (Supplementary Tables 9 and 10).

**Table 5:** Grain yield and other agronomic traits of selected (top 15 and worst 5) early maturing maize inbred lines artificially inoculated with *Exserohilum turcicum* at Ikenne and Ile-Ife in 2017 and Ikenne, Ile-Ife, and Zaria in 2018.

Entry	Genotype	GYLD	DA	DS	ASI	PHT	EHT	HUSK	RL	SL	PASP	EASP	EROT	EPP	BI
76	TZEI 135	2490	54.9	55.7	0.9	123.3	54.1	3.0	1.4	0.1	3.0	3.0	4.6	0.9	7.0
24	TZEI 60	2060	60.2	61.3	1.1	151.3	73.4	2.0	0.4	0.8	2.0	3.0	2.5	0.8	7.0
56	TZEI 14	2131	58.3	60.4	2.2	102.2	47.1	2.0	0.5	0.4	3.0	3.0	1.5	0.8	6.0
68	TZEI 124	2104	54.9	56.7	1.8	146.2	63.7	3.0	1.1	0.0	4.0	3.0	2.6	0.8	5.0
42	TZEI 98	2269	56.3	57.3	1.0	121.4	49.8	3.0	0.2	0.7	4.0	4.0	4.9	0.9	5.0
21	TZEI 56	1564	53.0	54.5	1.4	127.9	48.2	3.0	0.7	0.4	4.0	4.0	3.7	0.9	4.0
83	TZEI 146	2080	57.3	58.1	0.8	113.6	51.7	3.0	1.0	0.3	5.0	4.0	3.4	0.9	4.0
35	TZEI 87	2131	54.7	55.8	1.1	132.0	53.3	2.0	1.5	0.8	4.0	4.0	3.7	0.9	4.0
80	TZEI 140	1902	59.5	61.6	2.1	131.9	47.4	4.0	0.9	0.0	4.0	4.0	2.8	0.9	4.0
59	TZEI 17	1966	56.5	56.7	0.4	86.6	34.1	4.0	0.8	0.0	4.0	4.0	4.4	0.8	4.0
92	TZEI 182	1144	54.3	55.4	1.3	92.9	39.9	4.0	0.9	0.7	5.0	5.0	4.8	0.8	0.0
89	TZEI 167	1338	58.4	60.8	2.4	92.0	37.1	4.0	0.2	−0.1	5.0	4.0	2.6	0.7	0.0
99	TZEI 223	1250	56.1	56.3	0.7	100.1	40.2	4.0	1.6	0.3	5.0	5.0	4.2	0.9	0.0
43	TZEI 103	1198	53.6	54.9	1.3	109.2	50.7	3.0	1.4	1.5	5.0	5.0	6.6	0.9	0.0
87	TZEI 163	1196	56.5	57.7	1.2	109.2	50.5	3.0	0.1	0.3	4.0	5.0	4.5	0.6	0.0
64	TZEI 118	476	56.4	58.2	1.8	65.6	35.8	5.0	1.0	−0.1	6.0	6.0	5.2	0.6	−5.0
93	TZEI 184	543	58.3	60.5	2.2	101.4	37.6	3.0	1.8	0.4	5.0	6.0	5.1	0.5	−5.0
19	TZEI 53	280	56.6	58.4	1.8	118.0	41.0	4.0	1.4	0.8	5.0	6.0	3.4	0.5	−5.0
30	TZEI 79	363	59.0	60.4	1.4	112.1	47.6	3.0	0.2	0.4	6.0	7.0	1.8	0.6	−7.0
81	TZEI 142	207	59.7	63.6	3.8	94.8	34.2	4.0	1.1	0.1	7.0	7.0	2.5	0.2	−11.0
	**LSD**	**480**	**1.5**	**1.6**	**0.8**	**13.2**	**8.0**	**0.9**	**1.1**	**0.8**	**0.7**	**0.7**	**2.3**	**0.1**	

LSD: least significant difference, GYLD: grain yield; DA: days to anthesis; DS: days to silking; ASI: anthesis-silking interval; PHT: plant height; EHT: ear height; HUSK: husk cover; RL: root lodging; SL: stalk lodging; PASP: plant aspect; EASP: Ear aspect; EROT: ear rot; EPP: ears per plot; BI: base index.

**Table 6:** Grain yield and other agronomic traits of selected (top 15 and worst 5) extra-early maturing maize inbred lines artificially inoculated with *Exserohilum turcicum* at Ikenne and Ile-Ife in and Ikenne, Ile-Ife and Zaria in 2018.

Entry	Genotype	GYLD	DA	DS	ASI	PHT	EHT	HUSK	RL	SL	PASP	EASP	EROT	EPP	BI
101	TZEEI 1	2913	53.2	53.8	0.6	148.3	57.2	2.0	0.5	1.1	4.0	4.0	5.7	0.8	7.0
119	TZEEI 21	2221	52.8	52.5	0.0	115.7	47.4	3.0	1.2	2.1	4.0	3.0	4.6	1.0	6.0
129	TZEEI 32	1877	53.9	54.7	0.7	143.5	59.0	3.0	1.2	1.2	4.0	4.0	3.7	0.9	6.0
112	TZEEI 13	2140	53.8	54.6	0.8	124.7	53.1	3.0	1.3	1.2	4.0	4.0	5.9	1.0	6.0
141	TZEEI 45	2042	55.1	56.3	1.3	107.3	43.2	3.0	0.5	0.6	4.0	3.0	2.2	0.9	6.0
113	TZEEI 14	1926	54.1	54.8	0.8	121.5	52.4	3.0	1.6	0.0	4.0	4.0	4.9	1.0	6.0
118	TZEEI 20	1980	55.7	56.6	0.9	122.5	54.2	3.0	0.1	0.5	4.0	3.0	2.0	0.8	6.0
121	TZEEI 24	1913	54.3	54.7	0.5	132.9	51.1	2.0	1.6	1.5	4.0	3.0	4.2	0.9	5.0
125	TZEEI 28	1990	57.9	59.0	1.3	158.2	63.3	3.0	1.3	0.6	4.0	4.0	3.0	0.7	5.0
103	TZEEI 3	2107	52.3	53.2	0.8	124.0	55.5	3.0	1.0	0.1	4.0	4.0	8.7	1.0	5.0
181	TZEEI 88	1286	58.0	59.1	1.0	102.6	43.7	3.0	0.8	0.7	5.0	4.0	2.4	0.8	1.0
149	TZEEI 53	1088	55.8	57.6	1.7	98.6	43.4	3.0	0.0	0.6	5.0	5.0	3.2	0.9	1.0
170	TZEEI 76	1218	53.9	55.3	1.8	111.2	47.5	3.0	0.5	0.7	5.0	4.0	4.6	0.8	1.0
179	TZEEI 86	935	53.6	54.4	1.0	102.7	36.1	3.0	0.5	1.3	5.0	4.0	0.4	0.9	0.0
116	TZEEI 18	1262	52.3	52.8	0.7	90.2	28.0	2.0	0.4	0.0	5.0	5.0	2.7	0.8	0.0
192	TZEEI 108	460	52.2	56.3	4.0	112.6	39.8	2.0	0.3	2.0	6.0	5.0	2.7	0.5	−6.0
138	TZEEI 42	428	56.8	58.5	1.5	112.8	38.1	4.0	0.5	0.1	6.0	6.0	2.3	0.4	−6.0
183	TZEEI 94	404	51.6	52.8	1.1	112.5	43.3	5.0	2.3	0.8	6.0	6.0	2.1	0.3	−7.0
176	TZEEI 83	369	51.8	54.3	2.5	105.6	34.7	5.0	1.4	0.7	6.0	7.0	1.9	0.4	−8.0
155	TZEEI 61	292	55.2	56.7	1.5	81.1	30.5	4.0	0.6	0.1	7.0	6.0	2.8	0.4	−8.0
	**LSD**	**439**	**1.3**	**1.4**	**0.8**	**13.8**	**9.3**	**0.7**	**1.1**	**0.9**	**0.6**	**0.7**	**2.1**	**0.2**	

LSD: least significant difference, GM: grand mean, CV (%): coefficient of variation, R^2^ (%) Coefficient of determination. GYLD: grain yield; DA: days to anthesis; DS: days to silking; ASI: anthesis-silking interval; PHT: plant height; EHT: ear height; HUSK: husk cover; RL: root lodging; SL: stalk lodging; PASP: plant aspect; EASP: Ear aspect; EROT: ear rot; EPP: ears per plot; BI: base index.

The classification of EM and EEM according to their resistance to NCLB, based on disease severity values across years and locations is presented in Supplementary Tables 11 and 12. Highly resistant inbreds were identified only within the EM group. In that category were inbred lines TZEI 60, TZEI 144, TZEI 122, TZEI 135, and TZEI 53. Six EM inbred lines were classified as highly susceptible. In contrast, there were no highly resistant EEM inbred lines. Within the EEM group, TZEEI 8, TZEEI 15, TZEEI 27, TZEEI 28, TZEEI 32, TZEEI 33, TZEEI 45, TZEEI 48, TZEEI 172, TZEEI 79, and TZEEI 99 were classified as resistant. Seventeen EEM inbred lines were classified as highly susceptible to *E. turcicum*.

For the EM group, there was a definite trend between grain yield and disease scores. Inbred lines with low disease severity scores (resistant) had high grain yield. For example, TZEI 135 and TZEI 60 which produced the highest grain yield, were also classified as highly resistant to the disease, while TZEI 79 and TZEI 142 with the lowest grain yield, were rated as highly susceptible to the disease (Table 5 and Supplementary Table 11). Contrarily, consistent trends in grain yield were not observed in the EEM group. For instance, TZEEI 1 had a significantly higher grain yield than those of other inbred lines in the EEM group but was moderately resistant to the *E. turcicum*. However, TZEEI 32 with a lower GYLD, was resistant to *E. turcicum* infection (Table 6 and Supplementary Table 12).

Grain yield had significant correlation coefficients with most measured traits (Table 7, Table 8). For both EM and EEM inbred lines, HUSK, PASP, EASP, and the three disease severity scores were negatively correlated with GYLD, whereas, PLHT, EHT, and EPP had positive correlations with GYLD. Furthermore, results of the G × Y × T interaction biplots provided valuable information. For example, EM inbred line TZEI 60 was outstanding in terms of grain yield, and also had desirable (low) scores for PASP, EASP, and TURC whereas EM inbred line TZEI 173 had higher scores for PASP, EASP, and TURC suggesting susceptibility to *E. turcicum* (Fig. 1a). Similar results were detected for EEM inbred lines (Fig. 1b). The G × Y × T biplot analysis identified EPP, PLHT, EASP, PASP, EROT (for EEM only), and TURC as important traits that significantly contributed to GYLD in both maturity groups. Four studied traits, EPP, EASP, PASP, and TURC, had significant regression and correlation coefficients for GYLD (Table 7, Table 8) and were used to compute the BI for each maturity group as follows:

$$BI=GYLD+r_{1}EPP-r_{2}EASP-r_{3}PASP-r_{4}TURC$$

**Table 7:** Correlation coefficients of grain yield and other agronomic traits of early maturing maize inbred lines artificially inoculated with *Exserohilum turcicum* at Ikenne and Ile-Ife in 2017 and Ikenne, Ile-Ife, and Zaria in 2018.

	GYLD	DA	DS	ASI	PHT	EHT	HUSK	RL	SL	PASP	EASP	EROT	EPP	TURC2WAI	TURC6WAI	TURC
Grain yield, Kgha^−1^	1	−0.13	−0.19	−0.25*	0.45^a^	0.48^a^	−0.30^a^	−0.03	−0.13	−0.71^a^	−0.83^a^	0.21*	0.65^a^	−0.26*	−0.27^a^	−0.28^a^
Days to anthesis		1	0.95^a^	0.31^a^	−0.21*	−0.18	−0.12	−0.18	−0.23*	0.10	0.18	−0.28^a^	−0.13	−0.02	−0.01	−0.01
Days to silking			1	0.57^a^	−0.24*	−0.23*	−0.10	−0.30^a^	−0.19	0.17	0.20*	−0.37^a^	−0.24*	0.05	0.07	0.07
ASI				1	−0.19	−0.25*	−0.02	−0.4^a^	−0.02	0.25*	0.23*	−0.41^a^	−0.41^a^	0.26*	0.25*	0.26^a^
Plant height (cm)					1	0.81^a^	−0.33^a^	0.10	0.17	−0.5^a^	−0.35^a^	−0.01	0.31^a^	−0.26*	−0.27^a^	−0.28^a^
Ear height (cm)						1	−0.4^a^	0.13	0.11	−0.54^a^	−0.38^a^	0.10	0.33^a^	−0.27^a^	−0.28^a^	−0.29^a^
Husk cover							1	0.10	−0.10	0.38^a^	0.32^a^	0.29^a^	−0.32^a^	0.12	0.22*	0.19
Root lodging								1	0.04	0.14	0.16	0.33^a^	0.06	−0.02	0.05	0.02
Stalk lodging									1	0.15	0.14	−0.07	−0.07	0.08	0.15	0.13
Plant aspect										1	0.73^a^	−0.12	−0.49^a^	0.50^a^	0.50^a^	0.54^a^
Ear aspect											1	0.01	−0.63^a^	0.30^a^	0.30^a^	0.32^a^
Ear rot												1	0.24*	−0.02	−0.06	−0.04
Ear per plot													1	−0.28^a^	−0.37^a^	−0.35^a^
TURC2WAI														1	0.83^a^	0.93^a^
TURC6WAI															1	0.97^a^
TURC																1

a Correlation is significant at the 0.01 level (2-tailed), GYLD: grain yield; DA: days to anthesis; DS: days to silking; ASI: anthesis-silking interval; PHT: plant height; EHT: ear height; HUSK: husk cover; RL: root lodging; SL: stalk lodging; PASP: plant aspect; EASP: Ear aspect; EROT: ear rot; EPP: ears per plot; BI: base index; TURC2WAI: Disease severity score two weeks after inoculation; TURC6WAI: Disease severity score six weeks after inoculation and TURC: Average disease severity score.

**Table 8:** Correlation coefficients of grain yield and other agronomic traits of extra-early maturing maize inbred lines artificially inoculated with *Exserohilum turcicum* at Ikenne and Ile-Ife in 2017 and Ikenne, Ile-Ife, and Zaria in 2018.

	GYLD	DA	DS	ASI	PHT	EHT	HC	RL	SL	PASP	EASP	Ear rot	EPP	TURC2WAI	TURC6WAI	TURC
Grain yield, Kgha^−1^	1	0.02	−0.09	−0.36^a^	0.67^a^	0.67^a^	−0.46^a^	0.13	−0.09	−0.91^a^	−0.84^a^	0.53^a^	0.67^a^	−0.52^a^	−0.61^a^	−0.61^a^
Days to anthesis		1	0.94^a^	0.02	0.02	0.16	−0.23*	−0.21	−0.17	−0.17	−0.08	−0.18	0.19	−0.17	−0.36^a^	−0.31^a^
Days to silking			1	0.34^a^	−0.01	0.12	−0.20	−0.24*	−0.19	−0.06	0.06	−0.26*	0.06	−0.04	−0.25*	−0.17
ASI				1	−0.09	−0.11	0.12	−0.12	−0.06	0.32^a^	0.39^a^	−0.26*	−0.36^a^	0.37^a^	0.32^a^	0.37^a^
Plant height (cm)					1	0.83^a^	−0.16	0.34^a^	−0.06	−0.65^a^	−0.48^a^	0.37^a^	0.24*	−0.39^a^	−0.37^a^	−0.39^a^
Ear height (cm)						1	−0.20	0.39^a^	−0.03	−0.68^a^	−0.50^a^	0.39^a^	0.34^a^	−0.42^a^	−0.44^a^	−0.46^a^
Husk cover							1	0.15	0.02	0.50^a^	0.55^a^	−0.19	−0.59^a^	0.18	0.32^a^	0.29^a^
Root lodging								1	0.27^a^	−0.13	−0.08	0.24*	−0.02	0.03	0.06	0.03
Stalk lodging									1	0.05	−0.07	0.04	−0.06	0.25*	0.09	0.14
Plant aspect										1	0.87^a^	−0.41^a^	−0.69^a^	0.54^a^	0.70^a^	0.68^a^
Ear aspect											1	−0.30^a^	−0.75^a^	0.46^a^	0.61^a^	0.59^a^
Ear rot												1	0.38^a^	−0.28^a^	−0.27^a^	−0.29^a^
Ear per plot													1	−0.58^a^	−0.65^a^	−0.61^a^
TURC2WAI														1	0.79^a^	0.91^a^
TURC6WAI															1	0.97^a^
TURC																1

a Correlation is significant at the 0.01 level (2-tailed), GYLD: grain yield; DA: days to anthesis; DS: days to silking; ASI: anthesis-silking interval; PHT: plant height; EHT: ear height; HUSK: husk cover; RL: root lodging; SL: stalk lodging; PASP: plant aspect; EASP: Ear aspect; EROT: ear rot; EPP: ears per plot; BI: base index; TURC2WAI: Disease severity score two weeks after inoculation; TURC6WAI: Disease severity score six weeks after inoculation and TURC: Average disease severity score.

**Fig. 1a:**
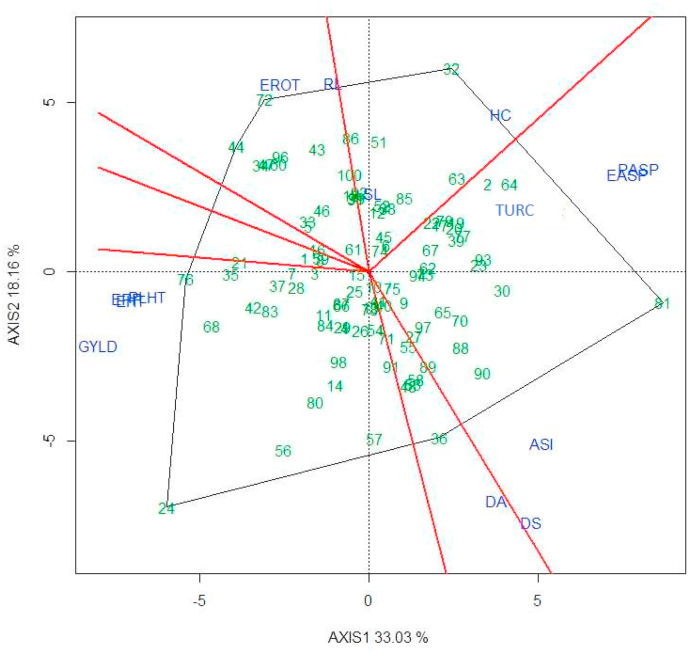
The ‘which-won-where’ of genotype by trait interaction of early maturing maize inbred lines artificially inoculated with *Exserohilum turcicum* at Ikenne and Ile-Ife during 2017 growing season and Ikenne, Ile-Ife and Zaria during 2018 growing seasons.

**Fig. 1b:**
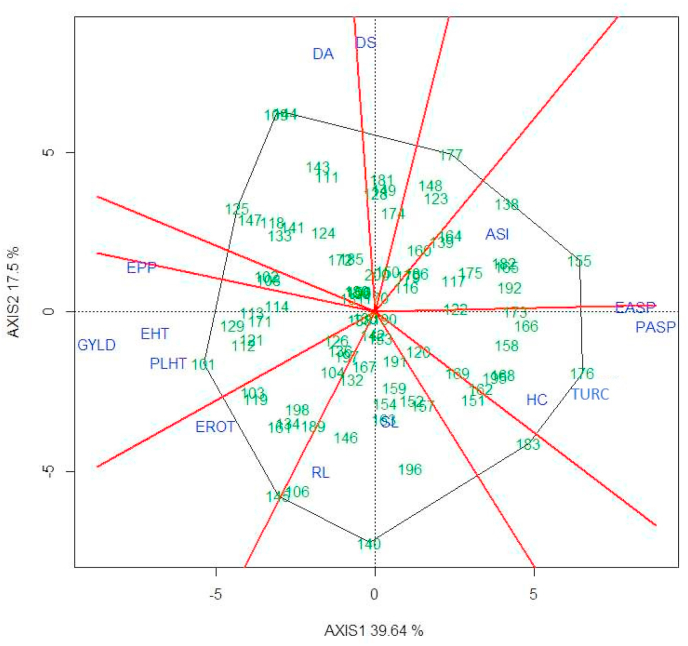
The ‘which-won-where’ of genotype by trait interaction of extra-early maturing maize inbred lines artificially inoculated with *Exserohilum turcicum* at Ikenne and Ile-Ife during 2017 growing season and Ikenne, Ile-Ife and Zaria during 2018 growing seasons.

The exact BI equations were:

(1) BI=GYLD+0.7EPP−0.8EASP−0.7PASP−0.3TURC

for EM, and

(2) BI=GYLD+0.7EPP−0.8EASP−0.9PASP−0.6TURC

for EEM

The 15 best performing EM (Table 3) and EEM (Table 4) inbred lines had BI values ranging from 0.0 to 7.0. The results indicated that the higher the BI, the higher the grain yield. Regression of GYLD on the BI of the 15 inbred lines resulted in equation Ŷ = 160.9x+1251.8, r^2^ = 0.86 for EM, and Ŷ = 187.1x + 1032.3, r^2^ = 0.84 for EEM.

Sequential path analysis produced similar results to those obtained in the G × Y × T biplots. For the EM inbred lines, EASP, ear rot, plant height, and days-to-silking were first order traits whose combined effects explained about 76% of the total variation in GYLD (Supplementary Figure 5). Of the five first-order traits, EASP had the highest direct effect on GYLD while plant height had the lowest. There were eight traits in the second order category. Of these, PASP had the highest indirect effects on GYLD through EASP. EPP also contributed indirectly to grain yield through EASP and ear rot while ear height had indirect effects through plant height. Disease severity (two weeks afer inoculation), stalk lodging, and disease severity (six weeks afer inoculation) were third-order traits. Disease severity (two weeks afer inoculation) contributed indirectly to GYLD through PASP, disease severity (six weeks afer inoculation) contributed indirectly to GYLD through EPP, anthesis silking interval, and ear height (Supplementary Figure 5).

Contrarily, for the EEM inbred lines PASP, days-to-silking, plant height, EASP, ear rot, and root lodging were first order traits, and explained about 90% of the total variation in GYLD (Supplementary Figure 6). Of the six first-order traits, PASP had the highest direct effect on GYLD followed by EASP while RL had the least effect. There were seven traits in the second order, namely EPP, ear height, disease severity (six weeks afer inoculation), husk cover, days-to-anthesis, anthesis-silking-interval, and stalk lodging. Of these traits, disease severity (six weeks afer inoculation) had significant indirect effects on GYLD through PASP and EASP. EPP also had indirect contributions to GYLD through PASP and EASP. Disease severity (two weeks afer inoculation) and TURC were the third-order traits. Disease severity (two weeks afer inoculation contributed indirectly to GYLD through all the second-order traits except days-to-anthesis and also had a significant indirect effect on grain yield, disease severity (six weeks afer inoculation), plant aspect, and ear aspect, whereas, TURC also contributed indirectly to GYLD through all the second-order traits except days-to-anthesis and husk cover (Supplementary Figure 6).

## 4 Discussion

Identification and selection of parental lines with high grain yield and high levels of resistance to NCLB, are critical for the development of hybrids with combined high yield potential and resistance to the disease (Sibiya et al., 2013). In maize, several traits of interest to the breeder are governed by additive gene action, that is, quantitatively inherited (Lindhout, 2002; Pswarayi and Vivek, 2008; Sibiya et al., 2013) and are passed on from parents to offsprings. In the present study, 100 each of EM and EEM maize inbred lines were evaluated in field trials with a view to identifying lines with high grain yield and stable resistance to NCLB. The inbred lines were evaluated at three locations belonging to different agro-ecologies of Nigeria during the 2017 and 2018 cropping seasons. The oubreak of NCLB had been observed in the test locations (Akinwale and Oyelakin, 2018). Therefore, the selected test locations were appropriate for our evaluations and facilitated the establishment of the pathogen and subsequent infection.

The morphological features of *E. turcicum* utilized in the present study were similar to those reported for the fungus (Tang et al., 2015; Carson, 2016). In order to identify an *E. turcicum* isolate for use in the field evaluations, the pathogenicity of seven *E. turcicum* isolates was evaluated in laboratory and screenhouse tests. It was important to establish the pathogenicity of these isolates since this was the first collection evaluated against EM and EEM maize germplasm of IITA. Although all the isolates established a pathogenic relationship with the two tested inbred lines, the most virulent isolate was NGIB16-13, and was selected for the multi-locations evaluations conducted in the present study. Therefore, the other isolates (NGIB16-2, NGIB16-3, NGIK16-5, NGIK16-11, NGIK16-12, and NGIF16-6) could also be used in screening for NCLB resistance in subsequent experiments. It is important to point out that using a single isolate in our evaluations had limitations. It is possible that some of the inbred lines possessed resistance to races that may be common in WCA and that were not tested in the present study. At least seven physiological races of *E. turcicum* have been described (Poland et al., 2011; Carson, 2016). Presently, it is unknown which races are prevalent in WCA. The races of *E. turcicum* are classified based on the maize germplasm for which they are virulent (Carson, 2016). In the near future, collaborations should be established with research groups possessing the differential maize germplasm set recommended for identification of *E. turcicum* races. In addition, the intense pressure to develop NCLB resistant germplasm prevented us from determining the race structure of *E. turcicum* in the sub-region. Therefore, we decided to use the most virulent *E. turcicum* isolate identified in our preliminary experiments using the two inbred lines, TZEEI 3 and TZEEI 30.

Each of the inoculation methods used in the preliminary screenings was successful and could be used for preliminary screening for resistance to NCLB. DLA permits the screening of a large number of germplasm as well as several strains of pathogens for rapid resistance identification prior to field evaluation (Aregbesola et al., 2019). The protocols used for the inoculum production as well as field inoculations were effective. The pathogen sporulated and colonized the sterile sorghum within 5 days of inoculation. The whorl of plants at 4 weeks after planting were well developed and could contain and retain the calibrated grains apportioned to each plant. The plant-to-plant inoculation method, which allowed each plant to be in close contact with a highly pathogenic *E. turcicum* isolate, was highly effective.

Our inoculation method also gave allowance for the progressive development of the disease as the symptoms spread from the point of inoculation to the upper leaves during the developmental stages. Therefore, the observed disease symptoms were considered to be incited by the inoculated *E. turcicum* isolate. Other field plant-to-plant inoculation methods for this pathosystem include atomizing spore suspensions (Ahangar et al., 2016), dropping into the whorl a spore suspension (150 ml) (Weems and Bradley, 2018) or ground symptomatic leaves collected the preceding season (Debela et al., 2017). Other researchers combined spore suspension and inoculated sorghum grains either simulatenously (Poland et al., 2011), or at five days interval (Bhat et al., 2017). All the methods mentioned are valuable but were not practical for our experiments because of the distance from the test locations, and logistic constraints. For example, transporting large quantities of liquid inoculum is complicated in our environments. Sometimes researchers inoculate twice during an experiment to ensure successful disease establishment. In our studies, inoculating only once proved to be effective. Although other researchers have utilized inoculated sorghum grains (Sermons and Balint-Kurti, 2018) supplemented with spore suspension, in our single-inoculation experiments we observed that the infected sorghum grains successfully established the disease. Obviously, natural high humidity of the environment eliminated the need to supplement the inoculum with spore suspension.

The resistance of EM and EEM maize inbred lines to *E. turcicum* evaluated in the current study were well established. EM inbred lines were in general, more resistant to NCLB compared to EEM inbred lines, regardless of year and location. Physiological differences between the two maturity groups have been reported. For instance, Akinwale et al. (2018) reported a differential association among EM and EEM under drought stress suggesting that under a particular stress, different mechanisms governed the reaction of inbred lines in each maturity group. Understanding stress tolerance mechanisms in EM and EEM inbred lines could improve production and productivity and reduce crop losses. Currently, the mechanisms of resistance among EM and EEM inbred lines are not well understood. It has been reported that *Ht2* and *Htn1* genes (Wisser et al., 2006) and QTL *qNCLB5.04* (Poland et al., 2011; Chen et al., 2016) contribute to NCLB resistance in maize plants. The presence of those genes/QTLs should be investigated in the evaluated set of EM and EEM inbreds. However, studies in Brazil revealed that monogenic resistance breaks down easily and could result in the emergence of new races of the pathogen (Ogliari et al., 2005). Pswarayi and Vivek (2008) and Sibiya et al. (2013) reported the role of additive gene action in resistance to NCLB. Therefore, the combination of *Ht* genes and polygenes would result in durable host plant resistance to the pathogen (Kim et al., 2012).

Immune maize plants (i.e. with no lesions or visible symptoms of NCLB) were not observed in the present study. However, a relatively high inoculum concentration was used for each plant and pathogen pressure was high. Even under this condition, there were some inbred lines with high levels of resistance, while others were tolerant to the pathogen. This calls for the need to upgrade the NCLB resistance level of some inbreds with high yield potential and desirable agronomic traits, but high susceptibility to NCLB. This could be done through the introgression of resistant genes from selected resistant inbred lines into susceptible EM and EEM lines. Such susceptible lines could produce higher yields if resistant genes are introgressed. As a major component of the disease triangle, the environment plays an important role in disease development (Vivek et al., 2010). Therefore, the significant (*P* < 0.05) G × E interactions observed in the present study suggested that some inbred lines had varying disease severity scores in the contrasting environments. The implication is that the expression of the resistance of the inbred lines could differ in contrasting environments.

An important objective of this study was to elucidate the inter-relationships among GYLD, disease score and other agronomic traits of EM and EEM inbred lines artificially inoculated with *E. turcicum*. It is not sufficient to consider a cultivar for cultivation based on one good agronomic trait but a combination of important traits. Selection of inbred lines with a combination of several desirable traits using the BI has been identified as a novel and useful approach (Yan and Kang, 2003; Oyekunle and Badu-Apraku, 2014). EASP, PASP, EPP, and TURC were identified as reliable secondary traits that contributed to grain yield of both EM and EEM inbred lines under NCLB infection. These traits have been identified by several authors as important traits associated with grain yield under multiple stresses, such as drought and low-nitrogen tolerance (Bänziger et al., 1999; Badu-Apraku et al., 2011; Oyekunle and Badu-Apraku, 2014; Talabi et al., 2017) and *Striga* infestation (Badu-Apraku and Akinwale, 2011; Akinwale et al., 2014; Badu-Apraku et al., 2018).

This is the first report on EM and EEM inbred lines for resistance/tolerance to NCLB. The resistant inbred lines identified in the present study should be classified into heterotic groups and crossed in hybrid combinations for the development of productive hybrids. It is important to screen inbred lines of other maize types and maturity groups in the IITA-MIP to identify NCLB resistant lines for the development of *E. turcicum* resistant hybrids for SSA. There is also the need to obtain a *E. turcicum* collections comprising isolates native to diverse regions of SSA for identification of tropical maize germplasm with durable resistance to NCLB.

## 5 Conclusions

In the present study, a protocol used for *E. turcicum* inoculation was found to be efficient for screening maize inbred lines for resistance to the pathogen. We developed a BI for selecting EM and EEM inbred lines for high yield potential, resistance to *E. turcicum*, and desirable agronomic traits. The careful testing in multiple locations during two cropping seasons allowed the identification of resistant inbred lines that could be used to develop hybrids with stable and durable resistance to the pathogen. The 53 EM and 55 EEM inbred lines identified as resistant/tolerant in the present study should be classified into heterotic groups and crossed in hybrid combinations for the development of productive hybrids for commercialization in SSA. The PASP, EASP, and EPP identified as secondary traits contributing directly to GYLD could improve the genetic gains from selection for increased GYLD under *E. turcicum* infection. In spite of the limitations of the study (i.e., use of a single isolate), the identification of *E. turcicum* resistant lines in the present study has set the stage for development of NCLB resistant EM and EEM hybrids for SSA as well as the improvement of tropical maize breeding populations for NCLB resistance. In the near future, the identified resistant germplasm should be challenged against other races of the pathogen. The availability of NCLB resistant inbred lines and hybrids would contribute to (i) increased maize production and productivity in WCA and (ii) improved yield, food security, and incomes of resource poor farmers in the sub-region.

## Declaration of competing interest

The authors declare that they have no known competing financial interests or personal relationships that could have appeared to influence the work reported in this paper
