# Supplementary material for: Identification of early and extra-early maturing tropical maize inbred lines resistant to Exserohilum turcicum in sub-Saharan Africa
Source: Crop Prot. 2021 Jan;139:105386. doi: 10.1016/j.cropro.2020.105386 (PMC7649949; doi:10.1016/j.cropro.2020.105386)
Supplement: Multimedia component 2 [file mmc2.docx]

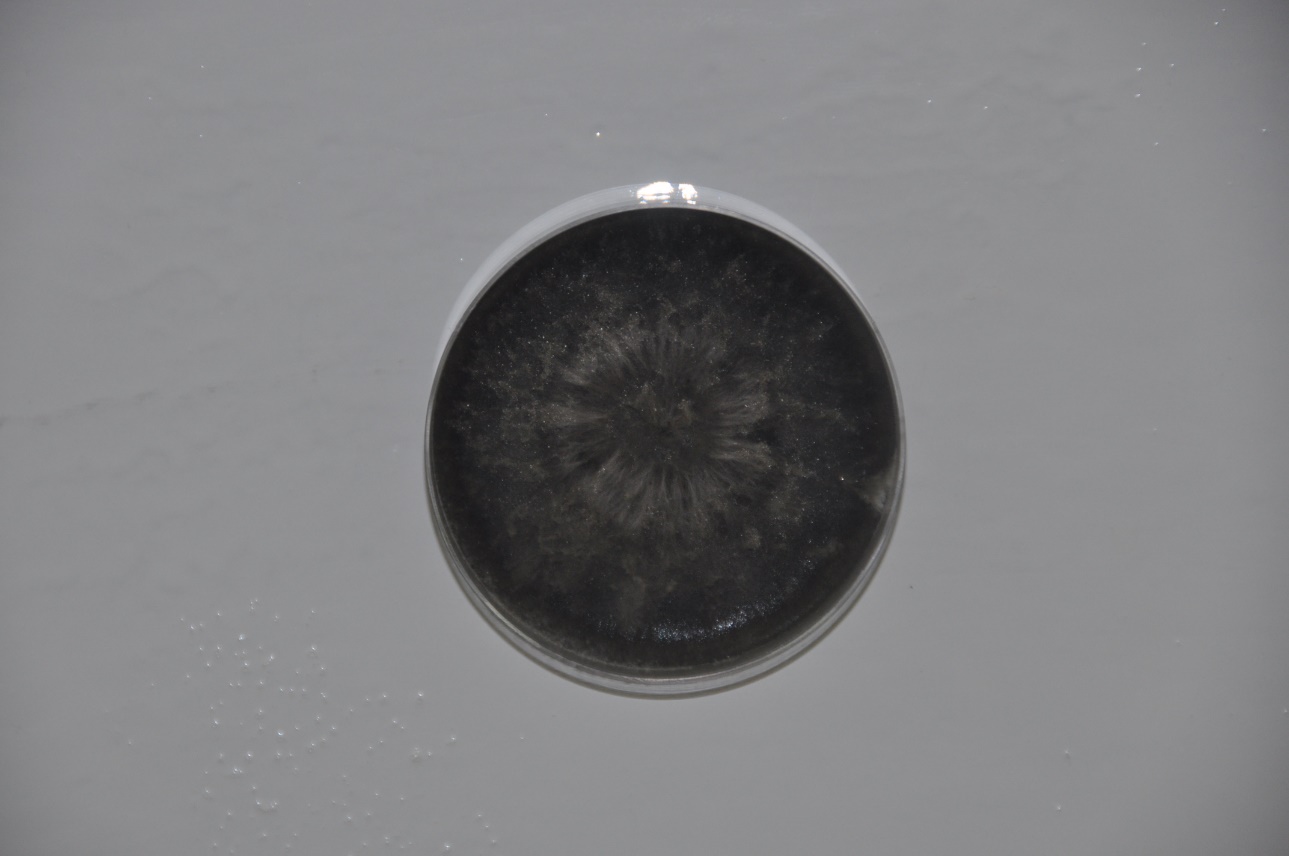

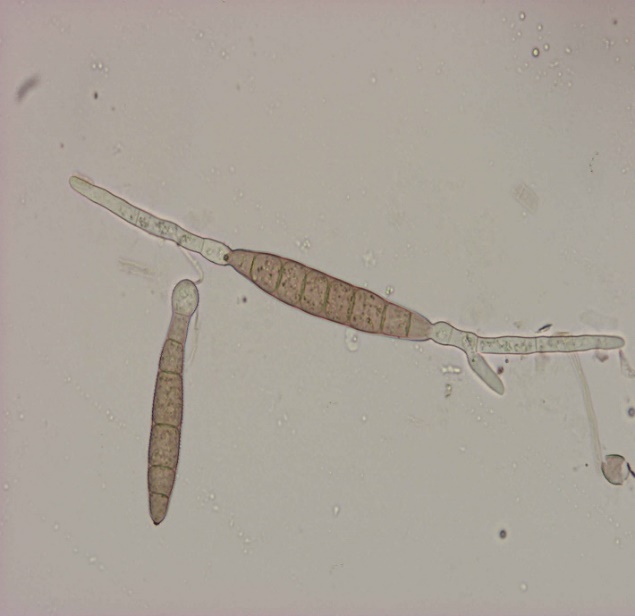

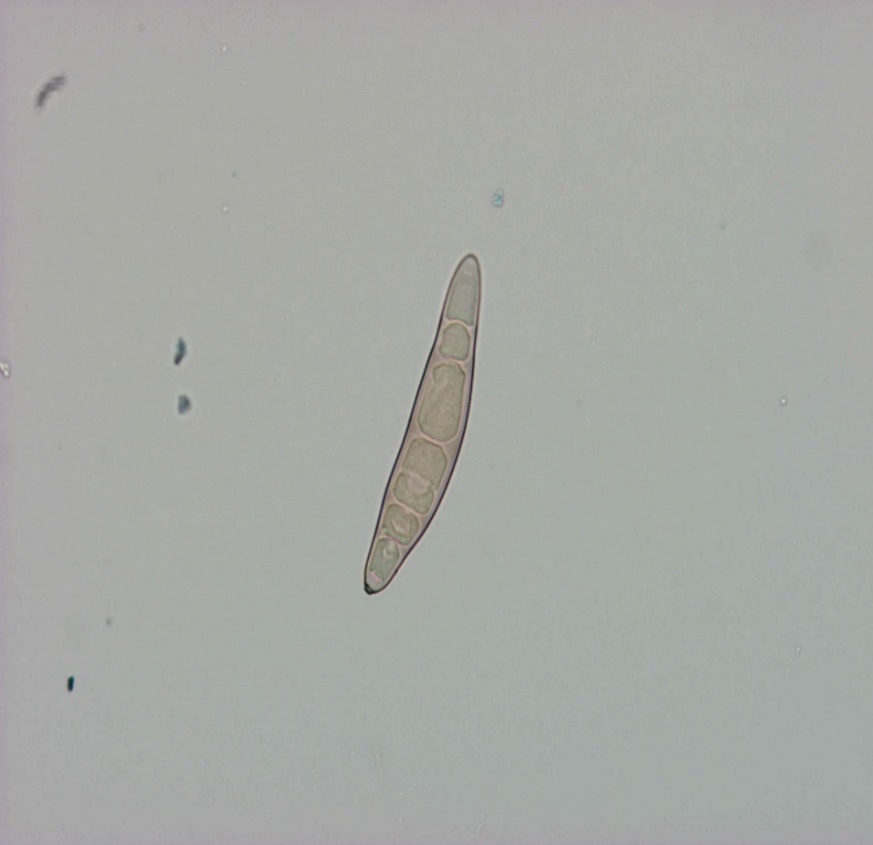


A

B

C

Supplementary Figure 1: 14-day-old colony of *Exserohilum turcicum* isolate NGIB16-13 on PDA

A: mycelia of *Exserohilum turcicum*, Scale bar = 30 µm, B: conidia of *Exserohilum turcicum*, with its characteristic hilum, Scale bar = 50 µm, C: germinating conidia and hyphae

Supplementary Figure 2: Pathogenicity test of *Exserohilum turcicum* strains using a detached leaf assay

DISEV: disease severity based on a rating scale of 1-5 (as earlier described), DAI: days after inoculation

Supplementary Figure 3: Response of maize inbred lines to pathogenicity test of *Exserohilum turcicum* using a detached leaf assay. (TURC: disease severity based on a rating scale of 1-5 (as earlier described), DAI: days after inoculation, TZEEI 30 and TZEEI 3 are extra-early inbred lines)

Supplementary Figure 4: Pathogenicity tests of *Exserohilum turcicum* isolate (NGIB16-13) using atomizing fungal suspensions and colonized sorghum grains methods (TURC: disease severity based on a rating scale of 1-5 (as earlier described), DAI: days after inoculation).

Supplementary Figure 5: Inter-trait relationships among early maize inbred lines inoculated with an *Exserohilum turcicum* isolate in different environments in Nigeria, during two cropping seasons.

Supplementary Figure 6: Inter-trait relationships among extra-early maize inbred lines inoculated with an *Exserohilum turcicum* isolate in different environments in Nigeria, during two cropping seasons.
