# Supplementary material for: Identification of early and extra-early maturing tropical maize inbred lines resistant to Exserohilum turcicum in sub-Saharan Africa
Source: Crop Prot. 2021 Jan;139:105386. doi: 10.1016/j.cropro.2020.105386 (PMC7649949; doi:10.1016/j.cropro.2020.105386)
Supplement: Multimedia component 3 [file mmc3.docx]

Supplementary Table 1: List of early maturing maize inbred lines artificially inoculated with *Exserohilum turcicum* at Ikenne and Ile-Ife in 2017 and Ikenne, Ile-Ife, and Zaria in 2018.

| **Entry no.** | **Kernel color** | **Genotype** | **Entry no.** | **Kernel color** | **Pedigree** | **Entry no.** | **Kernel color** | **Pedigree** | **Entry no.** | **Kernel color** | **Pedigree** | **Entry no.** | **Kernel color** | **Pedigree** |
| --- | --- | --- | --- | --- | --- | --- | --- | --- | --- | --- | --- | --- | --- | --- |
| 1 | White | TZEI 1 | 21 | White | TZEI 56 | 41 | White | TZEI 94 | 61 | Yellow | TZEI 24 | 81 | Yellow | TZEI 142 |
| 2 | White | TZEI 2 | 22 | White | TZEI 57 | 42 | White | TZEI 98 | 62 | Yellow | TZEI 25 | 82 | Yellow | TZEI 143 |
| 3 | White | TZEI 4 | 23 | White | TZEI 59 | 43 | White | TZEI 103 | 63 | Yellow | TZEI 115 | 83 | Yellow | TZEI 146 |
| 4 | White | TZEI 5 | 24 | White | TZEI 60 | 44 | White | TZEI 144 | 64 | Yellow | TZEI 118 | 84 | Yellow | TZEI 159 |
| 5 | White | TZEI 30 | 25 | White | TZEI 69 | 45 | White | TZEI 168 | 65 | Yellow | TZEI 119 | 85 | Yellow | TZEI 160 |
| 6 | White | TZEI 31 | 26 | White | TZEI 71 | 46 | White | TZEI 180 | 66 | Yellow | TZEI 120 | 86 | Yellow | TZEI 161 |
| 7 | White | TZEI 32 | 27 | White | TZEI 74 | 47 | White | TZEI 187 | 67 | Yellow | TZEI 122 | 87 | Yellow | TZEI 163 |
| 8 | White | TZEI 33 | 28 | White | TZEI 75 | 48 | White | TZEI 189 | 68 | Yellow | TZEI 124 | 88 | Yellow | TZEI 165 |
| 9 | White | TZEI 35 | 29 | White | TZEI 76 | 49 | White | TZEI 269 | 69 | Yellow | TZEI 126 | 89 | Yellow | TZEI 167 |
| 10 | White | TZEI 39 | 30 | White | TZEI 79 | 50 | White | TZEI 270 | 70 | Yellow | TZEI 127 | 90 | Yellow | TZEI 170 |
| 11 | White | TZEI 40 | 31 | White | TZEI 80 | 51 | Yellow | TZEI 8 | 71 | Yellow | TZEI 128 | 91 | Yellow | TZEI 173 |
| 12 | White | TZEI 45 | 32 | White | TZEI 82 | 52 | Yellow | TZEI 9 | 72 | Yellow | TZEI 130 | 92 | Yellow | TZEI 182 |
| 13 | White | TZEI 46 | 33 | White | TZEI 83 | 53 | Yellow | TZEI 11 | 73 | Yellow | TZEI 131 | 93 | Yellow | TZEI 184 |
| 14 | White | TZEI 47 | 34 | White | TZEI 86 | 54 | Yellow | TZEI 12 | 74 | Yellow | TZEI 132 | 94 | Yellow | TZEI 185 |
| 15 | White | TZEI 49 | 35 | White | TZEI 87 | 55 | Yellow | TZEI 13 | 75 | Yellow | TZEI 134 | 95 | Yellow | TZEI 186 |
| 16 | White | TZEI 50 | 36 | White | TZEI 88 | 56 | Yellow | TZEI 14 | 76 | Yellow | TZEI 135 | 96 | Yellow | TZEI 219 |
| 17 | White | TZEI 51 | 37 | White | TZEI 89 | 57 | Yellow | TZEI 15 | 77 | Yellow | TZEI 136 | 97 | Yellow | TZEI 220 |
| 18 | White | TZEI 52 | 38 | White | TZEI 90 | 58 | Yellow | TZEI 16 | 78 | Yellow | TZEI 138 | 98 | Yellow | TZEI 222 |
| 19 | White | TZEI 53 | 39 | White | TZEI 91 | 59 | Yellow | TZEI 17 | 79 | Yellow | TZEI 139 | 99 | Yellow | TZEI 223 |
| 20 | White | TZEI 54 | 40 | White | TZEI 93 | 60 | Yellow | TZEI 23 | 80 | Yellow | TZEI 140 | 100 | Yellow | TZEI 230 |

Supplementary Table 2: List of extra-early maturing maize inbred lines artificially inoculated with *E*xserohilum *turcicum* at Ikenne and Ile-Ife during 2017 growing season and Ikenne, Ile-Ife, and Zaria during 2018 growing seasons.

| **Entry no.** | **Kernel color** | **Genotype** | **Entry no.** | **Kernel color** | **Pedigree** | **Entry no.** | **Kernel color** | **Pedigree** | **Entry no.** | **Kernel color** | **Pedigree** | **Entry no.** | **Kernel color** | **Pedigree** |
| --- | --- | --- | --- | --- | --- | --- | --- | --- | --- | --- | --- | --- | --- | --- |
| 101 | White | TZEEI 1 | 121 | White | TZEEI 24 | 141 | White | TZEEI 45 | 161 | Yellow | TZEEI 67 | 181 | Yellow | TZEEI 88 |
| 102 | White | TZEEI 2 | 122 | White | TZEEI 25 | 142 | White | TZEEI 46 | 162 | Yellow | TZEEI 68 | 182 | Yellow | TZEEI 89 |
| 103 | White | TZEEI 3 | 123 | White | TZEEI 26 | 143 | White | TZEEI 47 | 163 | Yellow | TZEEI 69 | 183 | Yellow | TZEEI 94 |
| 104 | White | TZEEI 4 | 124 | White | TZEEI 27 | 144 | White | TZEEI 48 | 164 | Yellow | TZEEI 70 | 184 | Yellow | TZEEI 95 |
| 105 | White | TZEEI 5 | 125 | White | TZEEI 28 | 145 | White | TZEEI 49 | 165 | Yellow | TZEEI 71 | 185 | Yellow | TZEEI 96 |
| 106 | White | TZEEI 6 | 126 | White | TZEEI 29 | 146 | White | TZEEI 50 | 166 | Yellow | TZEEI 72 | 186 | Yellow | TZEEI 97 |
| 107 | White | TZEEI 7 | 127 | White | TZEEI 30 | 147 | White | TZEEI 51 | 167 | Yellow | TZEEI 73 | 187 | Yellow | TZEEI 98 |
| 108 | White | TZEEI 8 | 128 | White | TZEEI 31 | 148 | White | TZEEI 52 | 168 | Yellow | TZEEI 74 | 188 | Yellow | TZEEI 99 |
| 109 | White | TZEEI 10 | 129 | White | TZEEI 32 | 149 | White | TZEEI 53 | 169 | Yellow | TZEEI 75 | 189 | Yellow | TZEEI 100 |
| 110 | White | TZEEI 11 | 130 | White | TZEEI 33 | 150 | White | TZEEI 54 | 170 | Yellow | TZEEI 76 | 190 | Yellow | TZEEI 101 |
| 111 | White | TZEEI 12 | 131 | White | TZEEI 34 | 151 | Yellow | TZEEI 9 | 171 | Yellow | TZEEI 78 | 191 | Yellow | TZEEI 102 |
| 112 | White | TZEEI 13 | 132 | White | TZEEI 36 | 152 | Yellow | TZEEI 58 | 172 | Yellow | TZEEI 79 | 192 | Yellow | TZEEI 108 |
| 113 | White | TZEEI 14 | 133 | White | TZEEI 37 | 153 | Yellow | TZEEI 59 | 173 | Yellow | TZEEI 80 | 193 | Yellow | TZEEI 109 |
| 114 | White | TZEEI 15 | 134 | White | TZEEI 38 | 154 | Yellow | TZEEI 60 | 174 | Yellow | TZEEI 81 | 194 | Yellow | TZEEI 113 |
| 115 | White | TZEEI 16 | 135 | White | TZEEI 39 | 155 | Yellow | TZEEI 61 | 175 | Yellow | TZEEI 82 | 195 | Yellow | TZEEI 115 |
| 116 | White | TZEEI 18 | 136 | White | TZEEI 40 | 156 | Yellow | TZEEI 62 | 176 | Yellow | TZEEI 83 | 196 | Yellow | TZEEI 148 |
| 117 | White | TZEEI 19 | 137 | White | TZEEI 41 | 157 | Yellow | TZEEI 63 | 177 | Yellow | TZEEI 84 | 197 | Yellow | TZEEI 157 |
| 118 | White | TZEEI 20 | 138 | White | TZEEI 42 | 158 | Yellow | TZEEI 64 | 178 | Yellow | TZEEI 182 | 198 | Yellow | TZEEI 158 |
| 119 | White | TZEEI 21 | 139 | White | TZEEI 43 | 159 | Yellow | TZEEI 65 | 179 | Yellow | TZEEI 86 | 199 | Yellow | TZEEI 172 |
| 120 | White | TZEEI 23 | 140 | White | TZEEI 44 | 160 | Yellow | TZEEI 66 | 180 | Yellow | TZEEI 87 | 200 | Yellow | TZEEI 179 |

Supplementary Table 3: Rating scale for NCLB severity at two weeks after inoculation (TURC2WAI) and six weeks after inoculation (TURC6WAI) according to Ramathani et al. (2011) with modifications.

| TURC2WAI/  TURC2WAF | Description |
| --- | --- |
| 1 | Complete resistance, no visible NCLB symptoms |
| 2 | Minor infection, one to few scattered lesions on lower leaves, covering 5% of leaf surface |
| 3 | Slight infection on lower leaves with scattered lesions, covering up to 20% of leaf surface |
| 4 | Low-to-moderate infection on lower leaves with few scattered lesions on lower leaves, covering 21 to 30% of leaf surface |
| 5 | Moderate infection, abundant lesions on lower leaves and few on middle leaves, covering 31 to 50 % of leaf surface |
| 6 | Moderate-to-high infection, abundant lesions on lower leaves and a few on middle leaves, covering 51 to 64% of leaf surface |
| 7 | Abundant and coalescing lesions on lower and middle leaves extending to upper leaves, covering 64 to 75% of leaf surface |
| 8 | Abundant and coalescing lesions on most leaves, covering 76 to 80% of leaf surface |
| 9 | Abundant and coalescing lesions on all leaves covering >80% of the leaf surface with some plants prematurely dead |

Note: lower leaves refer to maize leaves present on the plant between the first and third vegetative growth stage, middle leaves are maize leaves between the leaves present at the third vegetative stage and the flower node, while upper leaves are leaves above the flower node

Supplementary Table 4: Plant aspect rating scale

| Plant aspect rating | Description: scoring is based on overall plant type (plant and ear heights), uniformity of plants, disease and insect damage and lodging |
| --- | --- |
| 1 | Excellent |
| 2 | Very good |
| 3 | Good |
| 4 | Satisfactory |
| 5 | Acceptable |
| 6 | Undesirable |
| 7 | Bad |
| 8 | Worse |
| 9 | Completely undesirable |

Plant aspect - The plant aspect scored at 10 weeks after planting

Supplementary Table 5: Ear aspect rating scale

| Ear aspect rating | Description: scoring based on characteristics such as disease and insect damage, ear size, grain ﬁlling, and uniformity of ears |
| --- | --- |
| 1 | Excellent, clean, uniform, large, and well-filled ears |
| 2 | Very good |
| 3 | Good |
| 4 | Satisfactory |
| 5 | Acceptable |
| 6 | Undesirable |
| 7 | Bad |
| 8 | Worse |
| 9 | Completely unacceptable, ears with undesirable features |

Ear aspect - Ear aspect rating at harvest, before collection of samples for moisture

Supplementary table 6: Husk cover rating scale

| Husk cover rating | Description: scoring is based on tightly arranged and extended beyond the ear tip |
| --- | --- |
| 1 | Excellent |
| 2 | Very good |
| 3 | Good |
| 4 | Satisfactory |
| 5 | Acceptable |
| 6 | Undesirable |
| 7 | Bad |
| 8 | Worse |
| 9 | Completely undesirable |

Supplementary Table 7: Analysis of Variance table for grain yield, disease severity scores and other agronomic traits of early maturing inbred lines artificially inoculated with *Exserohilum turcicum* at Ikenne and Ile-Ife in 2017 and Ikenne, Ile-Ife, and Zaria in 2018.

| **SV** | **DF** | **GYLD** | **DA** | **DS** | **ASI** | **PLHT** | **EHT** | **HUSK** | **RL** | **SL** | **PASP** | **EASP** | **EROT** | **EPP** | **TURC2WAI** | **TURC6WAI** | **TURC** |
| --- | --- | --- | --- | --- | --- | --- | --- | --- | --- | --- | --- | --- | --- | --- | --- | --- | --- |
| ENV | 4 | 31124810.2** | 303.5** | 124.7** | 65.4** | 151.8** | 6698.1** | 431.9** | 173.8** | 50.1** | 64.6** | 160.3** | 1781.2** | 2.3** | 72.3** | 61.8** | 177.8** |
| Rep | 1 | 633627.6 | 4.4 | 15.3 | 2.3 | 137.3 | 346.2 | 5.2 | 0.1 | 0.0 | 5.6 | 4.4 | 0.9 | 0.6 | 1.4 | 1.1 | 1.2 |
| Block (Rep) | 40 | 294938.1 | 2.4 | 4.1 | 0.8 | 118.5 | 62.6 | 0.6 | 1.5 | 0.9 | 0.5 | 0.6 | 8.7 | 0.0 | 0.5 | 0.6 | 0.4 |
| Genotype | 100 | 1678714.0** | 26.9** | 36.4** | 3.7** | 926.5** | 218.7** | 1.5** | 4.3** | 2.1** | 3.5** | 3.6** | 25.0** | 0.1** | 2.9** | 6.7** | 7.3** |
| G x E | 400 | 743734.7** | 6.6** | 8.7** | 1.3** | 223.7** | 83.0** | 1.8** | 3.1** | 1.3** | 1.6** | 1.6** | 13.9** | 0.1** | 0.8** | 1.7** | 1.0** |
| Error | 455 | 298144.8 | 2.8 | 3.2 | 0.8 | 135.3 | 49.0 | 0.6 | 1.7 | 0.9 | 0.6 | 0.6 | 6.9 | 0.0 | 0.5 | 0.8 | 0.4 |
| GM |  | 1,287 | 56.7 | 58 | 1.4 | 107 | 43.3 | 3.4 | 0.8 | 0.5 | 4.7 | 4.7 | 3.8 | 75.2 | 3.5 | 4.3 | 3.4 |
| CV (%) |  | 42 | 3 | 3 | 66 | 11 | 16 | 23 | 172 | 200 | 17 | 12 | 69 | 22 | 20 | 21 | 20 |
| R^2^ (%) |  | 82 | 86 | 86 | 78 | 84 | 83 | 90 | 76 | 71 | 82 | 86 | 84 | 79 | 83 | 83 | 91 |

SV: source of variation, DF: degree of freedom, GYLD: grain yield; DA: days to anthesis; DS: days to silking; ASI: anthesis-silking interval; PHT: plant height; EHT: ear height; HUSK: husk cover; RL: root lodging; SL: stalk lodging; PASP: plant aspect; EASP: Ear aspect; EROT: ear rot; EPP: ears per plot; TURC2WAI: Disease severity score two weeks after inoculation; TURC6WAI: Disease severity score six weeks after inoculation and TURC: Average disease severity score, BI: base index, GM: grand mean, CV (%): coefficient of variation, R^2^(%): Coefficient of determination.

Supplementary Table 8: Analysis of Variance table for grain yield, disease severity scores and other agronomic traits of extra-early maturing inbred lines artificially inoculated with *Exserohilum turcicum* at Ikenne and Ile-Ife in 2017 and Ikenne, Ile-Ife, and Zaria in 2018.

| **SV** | **DF** | **YIELD** | **DA** | **DS** | **ASI** | **PLHT** | **EHT** | **HUSK** | **RL** | **SL** | **PASP** | **EASP** | **EROT** | **EPP** | **TURC2WAI** | **TURC6WAI** | **TURC** |
| --- | --- | --- | --- | --- | --- | --- | --- | --- | --- | --- | --- | --- | --- | --- | --- | --- | --- |
| ENV | 4 | 22974528.5** | 394.2** | 244.7** | 96.0** | 9153.5** | 15659.7** | 604.7** | 258.6** | 136.0** | 57.9** | 226.4** | 1265.8** | 3.1** | 78.8** | 88.5** | 205.7** |
| Rep | 1 | 595031.1 | 18.3 | 23.9 | 0.1 | 1782.3 | 218.0 | 0.3 | 2.2 | 1.7 | 0.4 | 0.3 | 2.4 | 0.1 | 11.9 | 2.8 | 6.1 |
| Block (Rep) | 40 | 166252.9 | 2.9 | 4.1 | 0.7 | 135.4 | 50.3 | 0.2 | 2.2 | 0.8 | 0.3 | 0.6 | 5.9 | 0.0 | 0.7 | 0.7 | 0.4 |
| Genotype | 100 | 1982559.1** | 36.7** | 39.9** | 3.9** | 1038.1** | 352.4** | 2.6** | 4.3** | 5.5** | 4.3** | 3.4** | 23.2** | 0.2** | 2.6** | 5.6** | 18.2** |
| ENV*Genotype | 400 | 689931.7** | 5.7** | 6.9** | 1.7** | 236.9** | 99.2** | 2.7** | 4.8** | 2.8** | 1.2** | 1.8** | 12.9** | 0.1** | 0.8** | 1.4** | 1.0** |
| Error | 455 | 298144.8 | 2.2 | 2.5 | 0.7 | 146.5 | 66.2 | 0.4 | 1.7 | 1.1 | 0.4 | 0.6 | 5.6 | 0.0 | 0.6 | 0.6 | 0.4 |
| GM |  | 1,296 | 54.3 | 55.4 | 1.2 | 116.1 | 45.9 | 3.5 | 1.1 | 0.9 | 5 | 4.6 | 3.6 | 0.7 | 4.2 | 5.0 | 3.7 |
| CV (%) |  | 39 | 3 | 3 | 72 | 10 | 18 | 17 | 121 | 113 | 13 | 17 | 66 | 23 | 18 | 16 | 17 |
| R^2^ (%) |  | 85 | 90 | 89 | 83 | 85 | 86 | 96 | 83 | 83 | 87 | 89 | 84 | 83 | 80 | 86 | 95 |

SV: source of variation, DF: degree of freedom, GYLD: grain yield; DA: days to anthesis; DS: days to silking; ASI: anthesis-silking interval; PHT: plant height; EHT: ear height; HUSK: husk cover; RL: root lodging; SL: stalk lodging; PASP: plant aspect; EASP: Ear aspect; EROT: ear rot; EPP: ears per plot; TURC2WAI: Disease severity score two weeks after inoculation; TURC6WAI: Disease severity score six weeks after inoculation and TURC: Average disease severity score, BI: base index, GM: grand mean, CV (%): coefficient of variation, R^2^(%): Coefficient of determination.

Supplementary Table 9: Base index of early maturing inbred lines artificially inoculated with *Exserohilum turcicum* at Ikenne and Ile-Ife in 2017 and Ikenne, Ile-Ife, and Zaria in 2018.

| **Entry** | **Genotype** | **BI** | **Entry** | **Genotype** | **BI** | **Entry** | **Genotype** | **BI** | **Entry** | **Genotype** | **BI** | **Entry** | **Genotype** | **BI** |
| --- | --- | --- | --- | --- | --- | --- | --- | --- | --- | --- | --- | --- | --- | --- |
| **76** | TZEI 135 | 7 | **60** | TZEI 23 | 2 | **25** | TZEI 69 | 1 | **52** | TZEI 9 | -1 | **79** | TZEI 139 | -2 |
| **24** | TZEI 60 | 7 | **11** | TZEI 40 | 2 | **86** | TZEI 161 | 1 | **12** | TZEI 45 | -1 | **73** | TZEI 131 | -3 |
| **56** | TZEI 14 | 6 | **47** | TZEI 187 | 2 | **5** | TZEI 30 | 0 | **51** | TZEI 8 | -1 | **77** | TZEI 136 | -3 |
| **68** | TZEI 124 | 5 | **29** | TZEI 76 | 2 | **71** | TZEI 128 | 0 | **27** | TZEI 74 | -1 | **20** | TZEI 54 | -3 |
| **42** | TZEI 98 | 5 | **28** | TZEI 75 | 2 | **26** | TZEI 71 | 0 | **36** | TZEI 88 | -1 | **22** | TZEI 57 | -3 |
| **21** | TZEI 56 | 4 | **4** | TZEI 5 | 2 | **66** | TZEI 120 | 0 | **18** | TZEI 52 | -1 | **65** | TZEI 119 | -3 |
| **83** | TZEI 146 | 4 | **29** | TZEI 269 | 2 | **92** | TZEI 182 | 0 | **94** | TZEI 185 | -1 | **39** | TZEI 91 | -3 |
| **35** | TZEI 87 | 4 | **72** | TZEI 130 | 2 | **89** | TZEI 167 | 0 | **6** | TZEI 31 | -2 | **32** | TZEI 82 | -3 |
| **80** | TZEI 140 | 4 | **33** | TZEI 83 | 1 | **99** | TZEI 223 | 0 | **62** | TZEI 25 | -2 | **63** | TZEI 115 | -4 |
| **59** | TZEI 17 | 4 | **55** | TZEI 13 | 1 | **43** | TZEI 103 | 0 | **45** | TZEI 168 | -2 | **23** | TZEI 59 | -4 |
| **57** | TZEI 15 | 4 | **54** | TZEI 12 | 1 | **87** | TZEI 163 | 0 | **88** | TZEI 165 | -2 | **2** | TZEI 2 | -5 |
| **34** | TZEI 86 | 3 | **16** | TZEI 50 | 1 | **46** | TZEI 180 | 0 | **74** | TZEI 132 | -2 | **64** | TZEI 118 | -5 |
| **7** | TZEI 32 | 3 | **91** | TZEI 173 | 1 | **48** | TZEI 189 | 0 | **90** | TZEI 170 | -2 | **93** | TZEI 184 | -5 |
| **96** | TZEI 219 | 3 | **15** | TZEI 49 | 1 | **10** | TZEI 39 | -1 | **97** | TZEI 220 | -2 | **19** | TZEI 53 | -5 |
| **1** | TZEI 1 | 3 | **53** | TZEI 11 | 1 | **9** | TZEI 35 | -1 | **40** | TZEI 93 | -2 | **30** | TZEI 79 | -7 |
| **37** | TZEI 89 | 3 | **78** | TZEI 138 | 1 | **100** | TZEI 230 | -1 | **17** | TZEI 51 | -2 | **81** | TZEI 142 | -11 |
| **3** | TZEI 4 | 3 | **13** | TZEI 46 | 1 | **75** | TZEI 134 | -1 | **31** | TZEI 80 | -2 | **69** | TZEI 126 | -11 |
| **84** | TZEI 159 | 3 | **61** | TZEI 24 | 1 | **85** | TZEI 160 | -1 | **67** | TZEI 122 | -2 | **82** | TZEI 143 | -12 |
| **44** | TZEI 144 | 3 | **8** | TZEI 33 | 1 | **41** | TZEI 94 | -1 | **38** | TZEI 90 | -2 | **95** | TZEI 186 | -12 |
| **14** | TZEI 47 | 2 | **98** | TZEI 222 | 1 | **58** | TZEI 16 | -1 | **70** | TZEI 127 | -2 | **50** | TZEI 270 | -13 |

BI: base index

Supplementary table 10: Base index of extra-early maturing inbred lines artificially inoculated with *Exserohilum turcicum* at Ikenne and Ile-Ife in 2017 and Ikenne, Ile-Ife, and Zaria in 2018.

| **Entry** | **Genotype** | BI | **Entry** | **Genotype** | BI | **Entry** | **Genotype** | BI | **Entry** | **Genotype** | BI | **Entry** | **Genotype** | BI |
| --- | --- | --- | --- | --- | --- | --- | --- | --- | --- | --- | --- | --- | --- | --- |
| **101** | TZEEI 1 | 7 | **198** | TZEEI 158 | 3 | **200** | TZEEI 179 | 1 | **191** | TZEEI 102 | -1 | **166** | TZEEI 72 | -5 |
| **119** | TZEEI 21 | 6 | **197** | TZEEI 157 | 3 | **163** | TZEEI 69 | 1 | **157** | TZEEI 63 | -1 | **168** | TZEEI 74 | -5 |
| **129** | TZEEI 32 | 6 | **124** | TZEEI 27 | 3 | **146** | TZEEI 50 | 1 | **152** | TZEEI 58 | -1 | **165** | TZEEI 71 | -6 |
| **112** | TZEEI 13 | 6 | **106** | TZEEI 6 | 3 | **180** | TZEEI 87 | 1 | **140** | TZEEI 44 | -1 | **192** | TZEEI 108 | -6 |
| **141** | TZEEI 45 | 6 | **199** | TZEEI 172 | 3 | **181** | TZEEI 88 | 1 | **117** | TZEEI 19 | -2 | **138** | TZEEI 42 | -6 |
| **113** | TZEEI 14 | 6 | **134** | TZEEI 38 | 2 | **149** | TZEEI 53 | 1 | **139** | TZEEI 43 | -2 | **183** | TZEEI 94 | -7 |
| **118** | TZEEI 20 | 6 | **189** | TZEEI 100 | 2 | **170** | TZEEI 76 | 1 | **169** | TZEEI 75 | -2 | **176** | TZEEI 83 | -8 |
| **121** | TZEEI 24 | 5 | **111** | TZEEI 12 | 2 | **179** | TZEEI 86 | 0 | **177** | TZEEI 84 | -2 | **155** | TZEEI 61 | -8 |
| **125** | TZEEI 28 | 5 | **130** | TZEEI 33 | 2 | **116** | TZEEI 18 | 0 | **164** | TZEEI 70 | -2 | **193** | TZEEI 109 | -8 |
| **103** | TZEEI 3 | 5 | **132** | TZEEI 36 | 1 | **154** | TZEEI 60 | 0 | **196** | TZEEI 148 | -3 | **110** | TZEEI 11 | -8 |
| **108** | TZEEI 8 | 5 | **172** | TZEEI 79 | 1 | **128** | TZEEI 31 | 0 | **175** | TZEEI 82 | -3 | **194** | TZEEI 113 | -8 |
| **171** | TZEEI 78 | 5 | **188** | TZEEI 99 | 1 | **156** | TZEEI 62 | 0 | **148** | TZEEI 52 | -3 | **115** | TZEEI 16 | -8 |
| **109** | TZEEI 10 | 5 | **143** | TZEEI 47 | 1 | **142** | TZEEI 46 | 0 | **123** | TZEEI 26 | -3 | **178** | TZEEI 182 | -9 |
| **145** | TZEEI 49 | 5 | **131** | TZEEI 34 | 1 | **159** | TZEEI 65 | 0 | **122** | TZEEI 25 | -3 | **127** | TZEEI 30 | -11 |
| **102** | TZEEI 2 | 5 | **153** | TZEEI 59 | 1 | **104** | TZEEI 4 | 0 | **151** | TZEEI 9 | -4 | **135** | TZEEI 39 | -11 |
| **147** | TZEEI 51 | 4 | **185** | TZEEI 96 | 1 | **190** | TZEEI 101 | -1 | **195** | TZEEI 115 | -4 | **137** | TZEEI 41 | -11 |
| **144** | TZEEI 48 | 4 | **136** | TZEEI 40 | 1 | **174** | TZEEI 81 | -1 | **162** | TZEEI 68 | -4 | **105** | TZEEI 5 | -13 |
| **114** | TZEEI 15 | 4 | **126** | TZEEI 29 | 1 | **120** | TZEEI 23 | -1 | **173** | TZEEI 80 | -5 | **107** | TZEEI 7 | -13 |
| **133** | TZEEI 37 | 4 | **167** | TZEEI 73 | 1 | **186** | TZEEI 97 | -1 | **158** | TZEEI 64 | -5 | **184** | TZEEI 95 | -13 |
| **161** | TZEEI 67 | 4 | **150** | TZEEI 54 | 1 | **160** | TZEEI 66 | -1 | **182** | TZEEI 89 | -5 | **187** | TZEEI 98 | -15 |

BI: base index

| **TURC^a^** | **Reaction** | **Early maturing maize genotypes** | **Proportion of genotypes (percentage)** |
| --- | --- | --- | --- |
| 1.0 – 2.4 | Highly resistant | TZEI 53, TZEI 60, TZEI 144, TZEI 122, TZEI 135 | 5 |
| 2.5 – 3.4 | Resistant | TZEI 103, TZEI 189, TZEI 32, TZEI 33, TZEI 40, TZEI 45, TZEI 46, TZEI 47, TZEI 5, TZEI 56, TZEI 69, TZEI 71, TZEI 75, TZEI 76, TZEI 83, TZEI 91, TZEI 93, TZEI 94, TZEI 124, TZEI 128, TZEI 130, TZEI 132, TZEI 134, TZEI 138, TZEI 139, TZEI 14, TZEI 146, TZEI 15, TZEI 159, TZEI 16, TZEI 163, TZEI 17, TZEI 182, TZEI 184, TZEI 220, TZEI 222, TZEI 223, TZEI 25 | 38 |
| 3.5 – 4.4 | Moderately resistant | TZEI 1, TZEI 168, TZEI 187, TZEI 269, TZEI 30, TZEI 31, TZEI 35, TZEI 39, TZEI 4, TZEI 50, TZEI 51, TZEI 52, TZEI 54, TZEI 57, TZEI 74, TZEI 80, TZEI 86, TZEI 87, TZEI 89, TZEI 90, TZEI 98, TZEI 11, TZEI 118, TZEI 12, TZEI 120, TZEI 127, TZEI 13, TZEI 131, TZEI 136, TZEI 140, TZEI 160, TZEI 161, TZEI 165, TZEI 167  TZEI 170, TZEI 173, TZEI 185, TZEI 219, TZEI 23, TZEI 230, TZEI 24, TZEI 8, TZEI 9 | 43 |
| 4.5 – 5.4 | Susceptible | TZEI 180, TZEI 2, TZEI 49, TZEI 59, TZEI 79, TZEI 88, TZEI 115, TZEI 119 | 8 |
| 5.5 - 9.0 | Highly susceptible | TZEI 82, TZEI 126, TZEI 142, TZEI 143, TZEI 186, TZEI 270. | 6 |

Supplementary Table 11: Reaction of early maturing maize inbred lines artificially inoculated with *Exserohilum turcicum* at Ikenne and Ile-Ife in 2017 and Ikenne, Ile-Ife, and Zaria in 2018.

**^a^** Average disease severity rating
